# The role of cognitive restraint savings and the safety of ketogenic weight loss interventions

**DOI:** 10.1590/1806-9282.20231505

**Published:** 2024-05-03

**Authors:** Jônatas de Oliveira

**Affiliations:** 1 Universidade de São Paulo, School of Medicine – São Paulo (SP), Brazil.

Achieving a caloric deficit through diets and meal plans requires various actions, including planning, consistency, and effective execution. These actions necessitate cognitive processing of information, decision-making, and choice^1^. Additionally, they require skills like inhibiting impulses, managing cravings and conflicts that arise with the desire to lose weight, challenging emotions associated with the body, and responding to environmental cues about food^2,3^. Many studies have identified factors that improve or impede adherence to dietary guidelines. The general consensus is that high adherence is crucial^4,5^. However, there are noteworthy dietary interventions, such as the ketogenic diet, that possess distinct characteristics.

The term “low-carb and ketogenic” encompasses various dietary possibilities, but I want to emphasize three distinctive nuances that are contradictory to the current discussion: (i) professionally prescribed low-carb and ketogenic diets; (ii) professionally prescribed food substitute options marketed as ketogenic diets; and (iii) self-imposed low-carb and ketogenic diets^6,7^. It is crucial to consider that studies that provide meals to participants might not reflect actual eating behavior, and their outcomes are based on highly regulated conditions. Thus, it appears that food substitutes may enhance both clinical studies and regular nutrition practice by offering a more practical solution. In particular, the inquiry surrounding these diets, despite their limitations and high cost, involves an aspect that is not often studied but is applied to all nutritional interventions: how much cognitive restraint individuals require to adhere to dietary recommendations^8–11^?

## THE ROLE OF COGNITIVE RESTRAINT

Cognitive restraint is defined as an effort made to eat less, but the way it is evaluated using the subscale “cognitive restraint” of the three factor eating questionnaire considers some cognitive distortions that subsidize the restrictions such as “I do not eat some foods because they make me fat”^12^. In nutritional treatment and future research, it is essential to consider how more prudent thinking that values nutrient importance can help make good choices and even impose food restrictions if required, without any cognitive distortions involved. We recently published a case study demonstrating that the utilization of food substitutes aided in the restoration of the resting metabolic rate in a woman who had a history of bulimia nervosa^13^. In this case study, a 36-year-old white woman with a history of obesity and bulimia nervosa who has had difficulty in losing and maintaining weight despite numerous dietary and pharmacological treatments was considered. There was a loss of 12 kg in 115 days, reaching 13.4 kg, with 11.4 kg of fat mass. The resting metabolic rate showed an increase of 79% in relation to the initial rate, reaching normal levels for the predictive equations and maintaining this level in the first-year follow-up^13^.

Opting for substitutes seems to eliminate several stages of food choice, wherein the notion of control over carbohydrate source foods is omnipresent during every meal and this control is solely in the hands of the individual^5^. The intervention successfully preserved lean mass and restored the basal metabolic rate to high levels. Prior to the intervention, efforts were directed toward behavioral nutrition to prevent the use of cognitive restriction as a solution. Simply lowering calorie intake was inadequate and it actually reinforced beliefs related to bulimia nervosa psychopathology^13^. Although it may seem to be an open question, the utilization of food substitutes offers additional safety measures when taking into account the stress that comes with self-imposed restrictions brought about by cognitive restraint^7,12^.

## IMPLEMENTATION OF KETOGENIC DIETS

It is essential not to overlook these factors as these individuals may need obesity treatments mostly due to reduced resting metabolic rates or high-fat mass that requires significant intervention time. Another issue neglected in many studies is the lack of pre-intervention eating behavior preparation. It is not worth to assume that a safe intervention will be effective for any individual. However, there will likely be an interaction between (i) eating history, (ii) cognitive-behavioral changes (such as disinhibition of eating behavior, increased fatphobic attitudes, intense food cravings, disconnection with internal signals, and changes in interoceptivity after many diets)^14–16^, and (iii) metabolic changes resulting from the length of time the individual follows the diet^16–19^.

While interventions using food substitutes may provide safer outcomes, they may not necessarily affect the behavioral and cognitive aspects reinforced by personal beliefs and the environment. Thus, further studies should concentrate on comprehensive screenings for an individual's history of eating disorders and behaviors that go beyond by simply identifying their risk or the presence of an eating disorder^20^. Successful screening should identify individuals who require behavioral therapy, specifically in the field of behavioral nutrition, to prepare them for dietary intervention and enable them to maintain a diet that aligns with the recommendations given post-intervention. As professionals, do we just recommend and track weight loss outcomes or do we also have an obligation to ensure the progress and involvement of individuals even after the completion of the diet? A weight-loss strategy that focuses solely on simplification or improved biochemical markers is inadequate for ensuring long-term outcomes for individuals struggling with obesity.
